# A Validation Study of the Web-Based Physical Activity Questionnaire Active-Q Against the GENEA Accelerometer

**DOI:** 10.2196/resprot.3896

**Published:** 2015-07-16

**Authors:** Stephanie Erika Bonn, Patrick Bergman, Ylva Trolle Lagerros, Arvid Sjölander, Katarina Bälter

**Affiliations:** ^1^ Department of Medical Epidemiology and Biostatistics Karolinska Institutet Stockholm Sweden; ^2^ Department of Sport Science Linnaeus University Kalmar Sweden; ^3^ Clinical Epidemiology Unit Department of Medicine Karolinska Institutet Stockholm Sweden; ^4^ Department of Endocrinology, Metabolism and Diabetes Karolinska University Hospital Huddinge Stockholm Sweden

**Keywords:** accelerometer, activity assessment, epidemiology, Internet, self report, validity

## Abstract

**Background:**

Valid physical activity assessment in epidemiological studies is essential to study associations with various health outcomes.

**Objective:**

To validate the Web-based physical activity questionnaire Active-Q by comparing results of time spent at different physical activity levels with results from the GENEA accelerometer and to assess the reproducibility of Active-Q by comparing two admissions of the questionnaire.

**Methods:**

A total of 148 men (aged 33 to 86 years) responded to Active-Q twice and wore the accelerometer during seven consecutive days on two occasions. Time spent on six different physical activity levels including sedentary, light (LPA), moderate (MPA), and vigorous (VPA) as well as additional combined categories of sedentary-to-light and moderate-to-vigorous (MVPA) physical activity was assessed. Validity of Active-Q was determined using Spearman correlation coefficients with 95% confidence intervals (CI) and the Bland-Altman method. Reproducibility was assessed using intraclass correlation coefficients (ICCs) comparing two admissions of the questionnaire.

**Results:**

The validity correlation coefficients were statistically significant for time spent at all activity levels; sedentary (*r*=0.19, 95% CI: 0.04-0.34), LPA (*r*=0.15, 95% CI: 0.00-0.31), sedentary-to-light (*r*=0.35, 95% CI: 0.19-0.51), MPA (*r*=0.27, 95% CI: 0.12-0.42), VPA (*r*=0.54, 95% CI: 0.42-0.67), and MVPA (*r*=0.35, 95% CI: 0.21-0.48). The Bland-Altman plots showed a negative mean difference for time in LPA and positive mean differences for time spent in MPA, VPA and MVPA. The ICCs of test-retest reliability ranged between *r*=0.51-0.80 for the different activity levels in Active-Q.

**Conclusions:**

More moderate and vigorous activities and less light activities were reported in Active-Q compared to accelerometer measurements. Active-Q shows comparable validity and reproducibility to other physical activity questionnaires used today.

## Introduction

Physical activity is a modifiable lifestyle factor, and while high activity levels are associated with decreased risks of non communicable diseases [1], inactivity is a leading global risk factor for mortality [2]. Both behaviors are important, yet complex to measure, as different types and intensities of activities may affect health differently. Valid assessment of physical activity in large epidemiological studies, as well as in intervention research, is therefore essential to study the associations with various health outcomes and to accurately measure physical activity, and changes in such, at different time points.

During the past decade, the use of Web-based instead of paper questionnaires has simplified data collection and improved data quality in large epidemiological studies [3]. Web-based data collection is also cost efficient due to such advantages as the use of automated data management systems for distribution of questionnaires and reminders and rapid return of high quality data obtained through implementation of, for example, automatic checks for erroneous or missing data at the time of response [4]. Selection bias has been of concern in Web-based data collection, but with increasing access to the Internet among populations worldwide, this problem has decreased substantially during the recent years [4]. Although physical activity questionnaires are feasible to use in large studies, they are prone to errors due to difficulties of recalling information, social desirability in answers and an inability to assess the complete spectrum of physical activity [5,6]. The validity of physical activity questionnaires used today varies, with most showing only moderate validity [7]. We have previously described and validated the Web-based physical activity questionnaire Active-Q with regards to total energy expenditure against doubly labeled water with good results (Spearman correlation coefficient: *r*=0.52, *P*<.001) [8]. However, another important aspect of physical activity behavior is time spent in different intensity levels, which the total energy expenditure does not convey.

Using accelerometers, movement can be objectively quantified and activities performed at different activity levels (eg light, moderate or vigorous) can be assessed. The devices are commonly worn around the waist or wrist, but wrist worn accelerometers have been shown to increase wear compliance and may thus decrease selection bias due to burden on study participants [9,10]. Therefore, to assess the validity of time spent at different intensity levels assessed with the Active-Q questionnaire, we collected accelerometer data using the wrist worn GENEA (gravity estimator of normal everyday activity) monitor [11], from 167 men. The primary aim of this study was to assess the validity of Active-Q against the GENEA with regards to time spent at sedentary, light, sedentary-to-light, moderate, vigorous and moderate-to-vigorous physical activity levels. The secondary aim was to assess the reproducibility of Active-Q by comparing results from two admissions of the questionnaire.

## Methods

### Study Design

Study participants were recruited from a large ongoing cohort study of men who underwent PSA (Prostate Specific Antigen) testing in Stockholm County, Sweden, from 2010 to 2012. All study participants enrolled in the cohort between March and May 2012 who had agreed to be contacted regarding additional studies, were eligible for and invited to participate in the VALTER study (VALidation against acceleromeTER).

In September 2012, 1348 men were emailed an invitation to participate in the VALTER study. Of these, 31 emails did not reach the recipient due to an invalid email address. Men who replied to the invitation were sent more detailed information about the study and were scheduled for an introductory meeting at Karolinska Institutet, Stockholm, Sweden. In total, 167 men agreed to participate. All participants were given both written and oral information about the study and signed an informed consent prior to participation.

**Figure 1:**
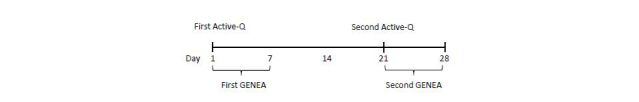
Timeline showing participants' responses to the first and second Active-Q questionnaire and when the first and second GENEA accelerometers were worn.

The study design is shown in Figure 1. Participants were enrolled in the study for a total of four weeks. On the first day of the study, the participants attended an introductory meeting at which they received the first GENEA accelerometer to wear during the following seven consecutive days. Participants also received the first Active-Q physical activity questionnaire via email on the evening of the same day. The questionnaire also included background questions on height, weight, birth year, education level and handedness. Individual user names and passwords served as identifiers for the questionnaire. After seven days, the accelerometer was returned to the research group via regular mail in a padded envelope with prepaid postage received during the introductory meeting. Three weeks later, on day 21 of the study, participants once again attended a meeting at a study site and were given a new GENEA accelerometer to wear for the following seven days before returning it via mail. They also received the second Active-Q questionnaire to respond to via email. All accelerometers were returned to study personnel at the end of each measurement period. An email reminder about the questionnaire was sent to participants who had not responded within a few days. Nevertheless, 84% responded the day of admission and a total of 96% had responded the following day.

Among the 167 men who agreed to participate, only participants with complete data from both questionnaire and accelerometer measurements were included in analysis. Participants were excluded due to drop out of the study (n=2) or due to erroneous accelerometer data from the first (n=3) or second (n=3) week of measurements. Further, men who reported to be left handed (n=11) were excluded from analysis as the accelerometer was worn on the left wrist. In total, data from 148 men were included in further analyses. As an incentive, all participating men received feedback from their accelerometer measurements approximately one month after the data collection was finished.

A subgroup of participants (n=22) partook in a calibration of the accelerometers. There were no differences in age, weight, height or BMI (body mass index) (*P*=.10 to .37) between men included in this subgroup and the whole study population. In the calibration, each participant wore two accelerometers on the same wrist while performing five predefined activities including: sitting, standing, and walking at a pace of 2, 3 and 4 mph. Each activity was performed for five minutes under the supervision of study personnel. Activities performed, and corresponding MET (metabolic equivalent task) values were retrieved from the Ainsworth Compendium of Physical Activities [12].

The study was approved by the Research Ethics Committee at Karolinska Institutet, Stockholm, Sweden.

### Active-Q

Active-Q is a Web-based, interactive physical activity questionnaire assessing habitual activity in adults (see Multimedia Appendix 1). It has previously been validated against doubly labeled water and has been described elsewhere [8]. Briefly, respondents report their usual activity during the past months within four different domains; daily occupation, transportation to and from daily occupation, leisure time activities, and regular sporting activities. The initial question to the means of transportation, leisure time activities and sporting activities are screening questions listing all the activities included in each domain. Only those activities selected by the participant in the screening are followed up with questions regarding frequency and duration, thereby, reducing the total number of questions each respondent needs to answer. An additional question on sleeping hours was also included, thus the questionnaire comprised 9 to 47 questions depending on previous answers and follow-up patterns. A screening question assessing working status (yes/no) preceded the questions of daily occupation and transportation. Participants reporting that they were not working did not get the questions concerning physical activity at work. All questions had predefined answers regarding frequency and duration. The additional question on sleeping hours, an addition of yoga and squash to the sporting activities, as these were frequently reported in an open response alternative to sporting activities in the previous study, and the screening question of working status were modifications made to the Active-Q after the previously published validation study [8].

All activities in Active-Q are linked to a corresponding MET value [12]. Activities with a MET value <1.5 are classified as sedentary, activities with a MET between 1.5 and <3 as light physical activity (LPA), activities with a MET of 3-6 as moderate physical activity (MPA) and activities with a MET >6 are classified as vigorous physical activity (VPA). Additional combined categories of activities classified as sedentary and light (sedentary-to-light activity) or moderate and vigorous physical activity (MVPA) were also created and included all activities with a MET <3 and ≥3, respectively. The total time reported in each category was calculated from Active-Q. An additional variable of total MET-h (reported time in hours for each specific activity multiplied by the activity’s MET-value) adjusted to a 24 hour period was also created by adding missing time or subtracting over-reported time to reach a total of 24 hours. Each hour added or subtracted was assumed to have a MET value of 2.0 as this was assumed to correspond to an average intensity of sitting, eating etc. (MET=1.5) and self caring, walking at home etc. (MET=2.5).

### GENEA Accelerometer

The GENEA accelerometer was developed by Unilever discover, UK and is manufactured and distributed by Activinsights Ltd., UK. It is a small (36 mm long x 30 mm wide x 12mm high, 16 gram) tri-axial accelerometer measuring vertical, anteroposterior and mediolateral movement at a rate of up to 80 Hz with a dynamic range of ±6g [11]. In the present study, acceleration was sampled at 40 Hz to decrease the amount of raw data while keeping a high enough sampling frequency to maintain accuracy. Study participants wore the accelerometer on their left wrist and were instructed to wear it continuously, but to remove it during water-based activities as this version of the accelerometer was not waterproof. Participants were provided with a diary to record all non-wear time (ie when the accelerometer was removed, for example during water activities). All recording of activities with a corresponding MET value >1.5 were corrected for in further analysis, including activities like swimming laps and water aerobics. For analysis in the present study, data from six complete days were extracted from each week of accelerometer measurements starting at midnight on the first day the accelerometer was worn. Only participants with complete data from six days of each measurement week were included in analysis. Results from the two measurement periods were thereafter combined and average daily times spent at different intensity levels were calculated using information from the total of 12 days.

**Figure 2:**
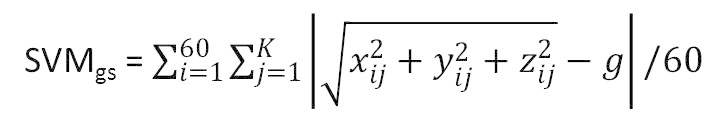
Equation of GENEA output per minute using the post processing software. K is the number of samples per second (K=40 in our study), and x_ij, y_ij, and z_ij is the acceleration along the three dimensions, respectively, at the j:th sample of the i:th second of the particular minute. g is set to 1.00 by default.

Using the same methods as Esliger et al. [11], the GENEA post processing software (version 1.2.1) was used to summarize the raw 40 Hz tri-axial data into a signal vector magnitude (SVM) (gravity subtracted) (SVM_gs_) and expressed as 1-minute epochs. Technically, for every minute the GENEA outputs SVM_gs_ defined by the equation given in Figure 2. The 1-minute epoch was obtained by multiplying each SVM_gs_ value with 60. Each SVM_gs_ value was further multiplied with 2 in order to make our SVM_gs_ values comparable to those reported by Esliger et al. [11], who used sampling frequency *K*=2 × 40 = 80 (*K* is the number of samples per second).

**Figure 3:**
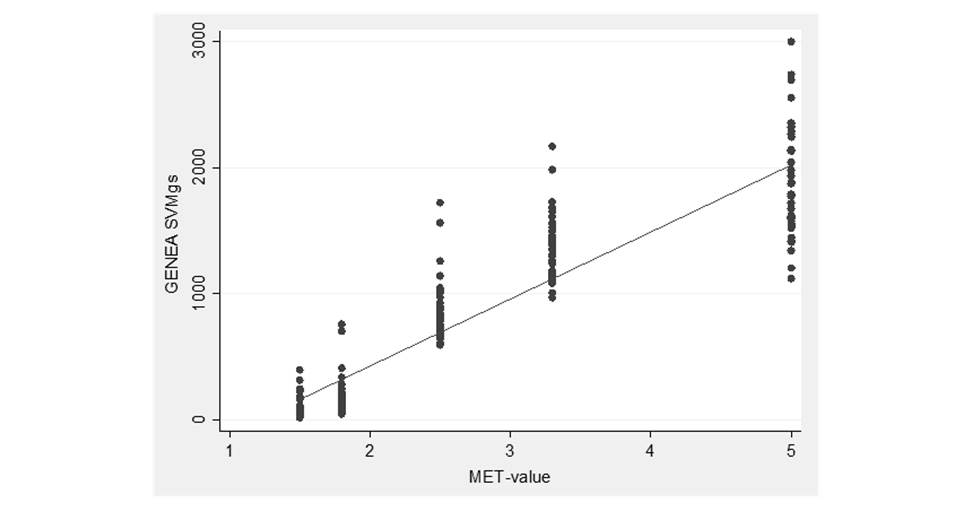
Scatter plot displaying MET-values of activities performed during the calibration (x-axis) and average GENEA-output in SVMgs (y-axis) for each specific activity, n=22 (44 measuring points).

Using data from the calibration of the accelerometers (Table 1), cut points specifically developed for the present study population of middle aged and older men were used to convert each SVM_gs_ value from the accelerometer into an activity level (sedentary, LPA, MPA or VPA). From each 5-minute interval of accelerometer measurements during the calibration, counts from the middle three minutes were extracted for analysis. The SVM_gs_ value for each participant and activity was then calculated and plotted against the corresponding MET value of the activity (Figure 3). We fitted a simple linear regression to these data, obtaining the fitted regression line SVM_gs_ = 529×MET -627 (y=529x – 627). The equation was thereafter used to determine cut points for SVM_gs_ corresponding to MET values 1.5, 3 and 6, for further classification of GENEA SVM_gs_ into sedentary (<1.5 MET), light (<3 MET), moderate (3-6 MET) or vigorous (>6 MET) activity levels. Combined categories of sedentary-to-light activity and MVPA were also created. Non-wear time recordings of activities with a MET value >1.5 were corrected for by subtracting time from the sedentary category and adding time to the LPA, MPA or VPA categories depending on the MET value of the reported activity.

**Table 1:** Mean GENEA SVM_gs_ (g·min) output for the five activities included in the calibration study.

Activity	MET Value	SVM_gs_ Mean^a^ (SD)
Sitting	1.5	105.2 (77.5)
Standing	1.8	167.1 (146.3)
Walking 3.2 km/h	2.5	826.0 (236.1)
Walking 4.8 km/h	3.3	1353.3 (246.2)
Walking 6.4 km/h	5.0	1875.3 (438.4)

^a^ Mean values are based on output from a total of 44 GENEA accelerometers.

### Statistical Analysis

Characteristics of study participants are presented as numbers and percentage, median or mean values with specified standard deviation (SD), total range and interquartile range (IQR). Differences between groups with regards to continuous and categorical variables were tested for using t-tests and chi-square tests, respectively.

Spearman correlation coefficients were used to assess the degree of association between time spent at sedentary, light, sedentary-to-light, moderate, vigorous or moderate-to-vigorous activity levels assessed with Active-Q and the accelerometers. Confidence intervals (CIs) for correlation coefficients were obtained using the bootstrap method [13]. In addition, Bland-Altman plots were used to assess systematic differences between the methods and as a graphical evaluation of the associations. The difference in time reported spent in each Activity category in Active-Q and measured with the accelerometer was plotted on the y-axis while the mean of the two methods was plotted on the x-axis. The limits of agreement, ±2 SD of the difference, provide a measure of variation. Weighted kappa statistics were estimated for quartiles of MPA, VPA and MVPA measured with Active-Q and GENEA.

For the reproducibility of Active-Q and GENEA, comparing results from the first and second measurements, intraclass correlation coefficients (ICCs) were computed using the ANOVA estimator. ICCs >70 and >90 were considered as moderate and strong, respectively, in line with the definitions used in a recent review of physical activity questionnaires [7]. Analyses were performed using STATA 12.1 (STATA Corporation, College Station, TX). The significance level was set to α = 0.05.

## Results

### Overview

Among the 148 men included in analyses, the mean age was 65.4 (SD 8.7) years and the mean BMI 25.7 (SD 2.9) kg/m^2^. Characteristics of study participants are presented in Table 2. The majority of men (57 %) reported that they were working full- or part-time. Participants were well educated and half of the men reported having studied at university level. The median response time of the first Active-Q responded to was 7 min and 19 sec.

Time spent at different activity levels estimated from the GENEA and Active-Q measurements are summarized in Table 3. The mean time spent sedentary and in LPA according to Active-Q was underestimated compared to GENEA, with a smaller difference between the methods for the combined category of sedentary-to-light activity. Correspondingly, the mean time spent in MPA, VPA and MVPA were overestimated in Active-Q compared to GENEA. While the average time spent in MPA was overestimated by approximately 70 minutes in Active-Q, the average time spent in VPA was overestimated by approximately 20 minutes, together corresponding well with the underestimation of time spent in LPA.

**Figure 4:**
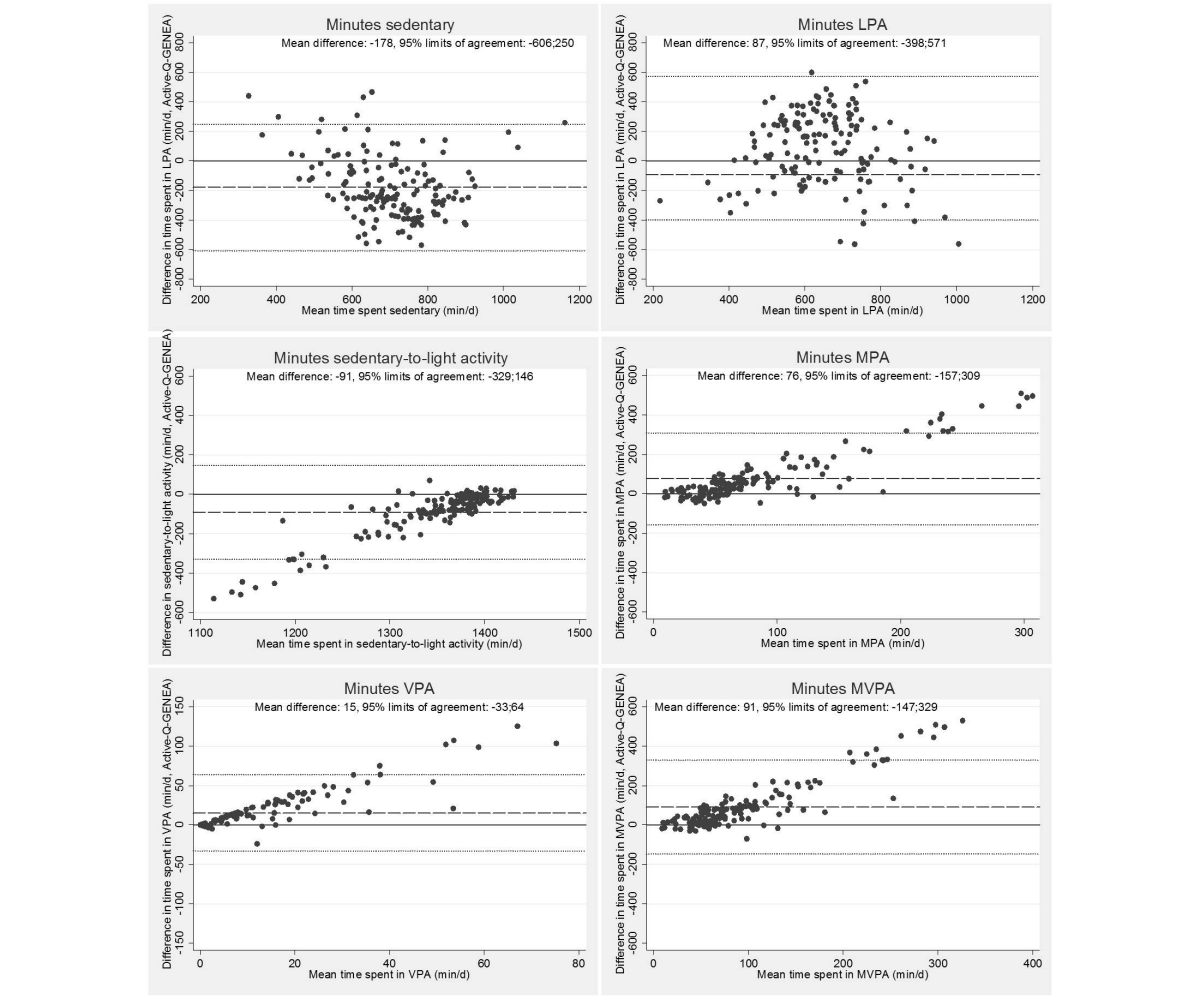
Bland-Altman plots illustrating differences in time spent sedentary, in light (LPA), sedentary-to-light, moderate (MPA), vigorous (VPA), and moderate-to-vigorous (MVPA) physical activity assessed with Active-Q and GENEA (y-axis) relative to the mean of the two methods (x-axis). Each point represents one study participant (n=148).

Spearman correlation coefficients with 95% confidence intervals (95% CI) for time at different activity levels are shown in Table 4. Bland-Altman plots comparing results between GENEA and Active-Q are displayed in Figure 4 **.** Statistically significant, but modest, correlations were found between estimates of time spent sedentary (*r*=0.19, 95% CI 0.04-0.34), in LPA (*r*=0.35, 95% CI 0.19-0.51), in sedentary-to-light activity (*r*=0.15, 95% CI 0.00-0.31), MPA (*r*=0.27, 95% CI 0.12-0.42) and MVPA (*r*=0.35, 95% CI 0.21-0.48) while the correlation for VPA was higher (*r*=0.54, 95% CI 0.42-0.67). The Bland-Altman plots illustrating the differences in time estimated with GENEA and Active-Q showed a negative mean difference for sedentary time and time in LPA and sedentary-to-light activity. Positive mean differences were seen for time spent in MPA, VPA and MVPA. The limits of agreement were wide for all activity levels. While no clear trend was seen for sedentary time or time spent in LPA, decreased accuracy at low levels of activity was seen for sedentary-to-light activity, and clear trends of decreased accuracy with increasing levels of time spent in MPA, VPA and MVPA were seen. A major contributing factor to the discrepancy in time spent in MVPA, as measured by Active-Q versus GENEA, was having reported working ≥20 h/week at a moderate or higher activity level. This was seen in all participants with a difference of >300 minutes between the methods (n=12). Among participants in the 75^th^ percentile of time spent in MVPA in Active-Q (>159 minutes), a high activity level at work, or performing household work, were the most common activities contributing time. Bicycling, spinning and/or skiing were reported by all participants having reported >100 min of VPA per day.

When dividing study participants into quartiles of time spent in MPA, VPA and MVPA assessed with GENEA and Active-Q, 32%, 46% and 33%, respectively, of participants were classified into the same quartile using both methods while 71%, 77% and 75%, respectively, were classified into the same or adjacent quartile. Results from weighted kappa statistics between the methods showed modest agreement, κ=0.16 (*P*=.004), κ=0.39 (*P*<.001) and κ=0.22 (*P*<.001) for MPA, VPA and MVPA, respectively.

ICCs comparing the first and second measurements of GENEA and Active-Q, respectively, are shown in Table 4. Overall, the GENEA accelerometer showed higher reproducibility compared to Active-Q for sedentary-to-light activity, MPA, VPA and MVPA. However, ICCs for sedentary time and LPA were low for the GENEA while high using Active-Q. The ICCs between different activity levels ranged from *r*=0.51-0.80 for Active-Q. Results for the two GENEA measurements showed higher ICCs for sedentary-to-light activity, MPA, VPA and MVPA ranging from *r*=0.76-0.78. The lowest ICC was found for sedentary time and LPA using the GENEA (*r*=0.25).

**Table 2:** Characteristics of study participants (n=148).

	Mean (SD)	Median	Min-Max	IQR^a^
Height, cm	179.2 (6.4)	179	165-198	175-183
Weight, kg	82.5 (11.0)	82	58-122	75-89
Age, years	65.4 (8.7)	66	33-86	61-71
BMI, kg/m^2^	25.7 (2.9)	25.4	19.6-35.6	23.5-27.5

^a^Interquartile range

**Table 3:** Results of time in minutes per day spent at light (LPA, <3 MET), moderate (MPA, 3-6 MET), vigorous (VPA, >6 MET), and moderate-to-vigorous (MVPA, ≥3 MET) physical activity levels assessed by GENEA and Active-Q (n=148).

		Mean (SD)	Median	Min-Max	IQR^a^
**First GENEA**					
	Sedentary	773 (234)	834	43-1107	708-926
	LPA	617 (234)	540	299-1340	468-664
	Sedentary + LPA	1390 (29)	1393	1269-1437	1379-1408
	MPA	47 (27)	46	3-165	30-59
	VPA	3 (6)	0	0-27	0-2
	MVPA	50 (29)	47	3-171	32-62
**Second GENEA**					
	Sedentary	804 (236)	853	83-1135	741-974
	LPA	589 (234)	533	268-1347	436-630
	Sedentary + LPA	1394 (31)	1399	1240-1438	1379-1415
	MPA	44 (29)	40	3-198	26-56
	VPA	3 (7)	0	0-61	0-3
	MVPA	47 (31)	42	3-201	26-62
**Average GENEA**					
	Sedentary	789 (186)	817	107-1116	676-941
	LPA	603 (185)	566	297-1286	463-696
	Sedentary + LPA	1392 (28)	1393	1255-1437	1380-1409
	MPA	46 (27)	44	3-182	31-56
	VPA	3 (6)	1	0-43	0-3
	MVPA	48 (28)	47	3-186	32-60
**First Active-Q**					
	Sedentary	611 (143)	579	360-1291	523-691
	LPA	690 (172)	708	83-1028	582-807
	Sedentary + LPA	1301 (123)	1339	849-1440	1281-1382
	MPA	121 (120)	84	0-555	51-135
	VPA	18 (26)	6	0-130	0-29
	MVPA	139 (123)	101	0-591	58-159
**Second Active-Q**					
	Sedentary	601 (142)	582	213-1351	516-683
	LPA	700 (191)	737	6-1109	596-826
	Sedentary + LPA	1301 (139)	1355	680-1428	1275-1390
	MPA	116 (123)	69	0-557	41-148
	VPA	22 (42)	9	0-289	0-29
	MVPA	139 (139)	85	12-760	50-165
**Average Active-Q**					
	Sedentary	606 (136)	578	338-1321	513-578
	LPA	695 (166)	716	73-1003	613-810
	Sedentary + LPA	1301 (120)	1346	872-1425	1265-1383
	MPA	119 (112)	72	11-527	72-160
	VPA	20 (31)	10	0-176	0-29
	MVPA	139 (120)	94	15-568	57-175

^a^Interquartile range

**Table 4:** Spearman correlation coefficients between time at different intensity levels and total MET-h in the first Active-Q questionnaire and time and total SVMgs from GENEA measurements during 12 days (n=148) and Intraclass correlation coefficients between the two Active-Q questionniares administered and between the two weeks of GENEA measurements (n=148).

	Spearman correlations		Intraclass correlations			
	Active-Q vs GENEA		Active-Q		GENEA	
	*r*	(95% CI)	*r*	(95% CI)	*r*	(95% CI)
Minutes/Day Sedentary	.19	(0.04-0.34)	.80	(0.74-0.86)	.25	(0.10-0.41)
Minutes/Day LPA	.15	(0.00-0.31)	.66	(0.57-0.75)	.25	(0.10-0.41)
Minutes/Day Sedentary + LPA	.35	(0.19-0.51)	.67	(0.58-0.76)	.78	(0.71-0.84)
Minutes/Day MPA	.27	(0.12-0.42)	.69	(0.60-0.77)	.76	(0.70-0.83)
Minutes/Day VPA	.54	(0.42-0.67)	.51	(0.39-0.63)	.77	(0.71-0.84)
Minutes/Day MVPA	.35	(0.21-0.48)	.67	(0.58-0.76)	.78	(0.71-0.84)

## Discussion

### Principal Findings

Our results from comparisons of Active-Q and the GENEA accelerometer show that Active-Q provides valid estimates of moderate and vigorous intensity activity although more time being active was reported in the questionnaire than assessed by the accelerometer. Active-Q showed acceptable reproducibility when comparing two admissions of the questionnaire.

### Comparison to Other Studies

Compared to accelerometer measurements, time spent at moderate and vigorous activity levels was overestimated in Active-Q. Over-reporting of physical activity is often due to misreporting of frequency, intensity and/or duration of activities [14]. Additional factors contributing to misreporting in general are social desirability [15] and memory bias, the latter particularly affecting older individuals who may have cognitive difficulties in recalling performed activities [16]. Although accuracy is important for determining clinically relevant levels of physical activity, the ranking ability of a questionnaire is often more important than the absolute measures in large epidemiological association studies. The observed correlations between Active-Q and the GENEA accelerometer are in line with previous studies of other physical activity questionnaires when compared to accelerometer measurements. In a recent systematic review [7], over 100 physical activity questionnaires were identified and the validity against objective criterion measures was moderate at best, with median correlation coefficients ranging from 0.25 to 0.41.

A commonly used physical activity questionnaire is the IPAQ (International Physical Activity Questionnaire) [17]. A recent review [18] summarized 23 validation studies of the short form of the IPAQ (IPAQ-SF) and showed that most studies presented weak correlations as compared to objective reference methods. Correlation coefficients between IPAQ-SF and accelerometer data ranged between 0.09 and 0.39 for total physical activity, with somewhat higher correlations for MPA and VPA. Similar to Active-Q, IPAQ-SF overestimated physical activity. In a more recent validation study of IPAQ, correlations between 0.50 and 0.61 were shown for time spent in MPA, VPA or MVPA when comparing questionnaire and accelerometer measurements [19]. However, the high correlations found may be explained by the fact that IPAQ was administered by telephone, and that participants reporting activities not captured by accelerometers (eg swimming and bicycling) were excluded. Dyrstad et al. [20], found correlations similar to the present study when comparing a self reported questionnaire and accelerometer measurements of time spent in MPA. Another recent publication of the validity of RPAQ (Recent Physical Activity Questionnaire) in ten European countries [21], showed similar correlation coefficients when comparing self reported and objectively measured MVPA among men. In the same study, also in line with the results of this study, time spent in MVPA was overestimated.

In addition to comparisons of validity with other existing questionnaires, it is important to remember the population for which the questionnaire is developed. Active-Q was originally developed for adults 18-45 years for use in a large cohort study [22], and has previously been validated with regard to energy expenditure, in a younger population than the present [8]. However, Active-Q is also in use in the cohort from which study participants for the present study, men with a median age of 66 years, were recruited. It is important to validate the questionnaire in a population that is representative of the cohort being studied, although this may limit the generalizability of results to the general population. A systematic review focusing on physical activity questionnaires validated in study populations with an average age >55 years showed diverging results [23]. However, the studies included covered different constructs than the present, such as physical activity level, energy expenditure or walking, making comparisons difficult. Nevertheless, in a recent study comparing questionnaire and accelerometer results, correlation coefficients for time spent in three different MET levels corresponding to LPA, MPA and VPA, were poor (*r*=0.05, 0.27 and 0.01, respectively) [24].

Results from the Bland-Altman plots, reflecting absolute differences between Active-Q and the GENEA accelerometer, showed that the difference between Active-Q and GENEA increased with increasing time spent in MPA, VPA and MVPA, similar to what has been seen in other studies [20]. Correspondingly for time spent in sedentary-to-light activity, the difference between the methods decreased with increasing time while no clear trends were seen for sedentary time and LPA, respectively. The difference between the methods could have several explanations including the inability of accelerometers to capture activities such as bicycling, spinning and swimming, which may contribute to lower levels of higher intensity activities being measured [25]. Further, static and non-ambulatory activities, such as carrying heavy loads and walking uphill, are not correctly captured by accelerometers [26]. Another explanation could be the different time periods assessed in Active-Q and with the GENEA accelerometer. Ideally, the reference method should reflect the same time period as the questionnaire under validation. However, while Active-Q assessed habitual physical activity during the past months prior to being filled out, the two weeks of accelerometer measurements were made after responding to the questionnaire, thus, not reflecting the same time period. The more long term recall in Active-Q, in contrast to the current accelerometer assessment, also limits the comparison since seasonal variability is not controlled for. That seasonal variability had an effect was indicated by the fact that winter sports contributed to the time in MVPA reported in Active-Q, although the data collection was made during the fall when these activities are unlikely to be performed and captured in accelerometer measurements. Therefore, our results of validity may be underestimated due to the study design. Preferably, the Active-Q should have been administered a few weeks after accelerometer measurements to reflect the same time period.

While our results show moderate reproducibility of Active-Q, few previous studies have reported test-retest reliability of time spent at different intensity levels, making comparisons difficult [7]. One study did nevertheless show ICCs of around 0.80 for a self-reported questionnaire developed for older adults [27]. However, the time between admissions of the questionnaires was only 1-2 weeks and shorter time periods between questionnaire assessments have been associated with higher reliability coefficients [7]. In the present study, the time between questionnaire assessments was three weeks in order to minimize differences due to true variation (eg seasonal changes) while still maintaining a long enough interval to decrease the risk of recalling the previous answers.

Although considered to be one of the best methods to objectively assess free living physical activity, accelerometers are not without limitations [25]. They are usually worn around the waist or wrist, both placements with their own strengths and limitations [28]. However, wrist worn accelerometers, as used in the present study, have been shown to increase wear compliance [9,10]. Although hip worn accelerometers have been shown to better classify activities into different intensity categories than wrist worn [29], the wrist worn GENEA has shown excellent validity [11]. A validation of the cut points developed by Esliger et al. [11] for GENEA worn on the left wrist found a modest accuracy of the intensity classification across a broad range of activities [30]. Another study has shown high accuracy in identifying specific activities [31]. The accelerometer output may differ between different populations and our calibration study resulted in higher cut points than those previously developed. Nevertheless, our cut points were developed using a small sample and a limited number of activities.

### Strengths and Limitations

In addition to the points of discussion raised in previous paragraphs, the present study has several strengths and limitations worth mentioning. First, the large sample size and the high compliance among participating men are important strengths. With some exceptions, most previous validation studies summarized in the review by Helmerhorst et al. [7] included fewer than 100 study participants in validity analysis while our study comprised almost 150 men. The high compliance and motivated study participants are further strengths to our study and made it possible to include 12 days of accelerometer measurements per individual. The number of days measured far exceeds the 3-5 days required to assess a daily estimate of the individual’s habitual activity, resulting in a valid ranking of participants [32]. It also exceeds the number of days commonly assessed in other validation studies using accelerometers [7]. Further, using an objective criterion measure with a different error structure compared to Active-Q also decreases the chance of correlated errors which otherwise may affect results [33].

### Conclusions

The present study shows that more moderate and vigorous time and fewer light activities are reported in Active-Q compared to the accelerometer measurements. Nevertheless, the questionnaire shows good ranking ability, and validity and reproducibility comparable to other physical activity questionnaires.

## Appendix

Multimedia Appendix 1 The Active-Q physical activity questionnaire.

## Abbreviations

BMI: body mass index
GENEA: gravity estimator of normal everyday activity
ICC: intraclass correlation coefficient
IPAQ: International Physical Activity Questionnaire
IPAQ-SF: International Physical Activity Questionnaire (Short Form)
LPA: light physical activity
MET: metabolic equivalent task
MET-h: metabolic equivalent task x hours reported for each specific activity
MPA: moderate physical activity
MVPA: moderate and vigorous physical activity
PSA: prostate specific antigen
RPAQ: Recent Physical Activity Questionnaire
SVMgs: signal vector magnitude (gravity subtracted)
VALTER: VALidation against acceleromeTER
VPA: vigorous physical activity
