# Supplementary material for: A Validation Study of the Web-Based Physical Activity Questionnaire Active-Q Against the GENEA Accelerometer
Source: JMIR Res Protoc. 2015 Jul 16;4(3):e86. doi: 10.2196/resprot.3896 (PMC4527001; doi:10.2196/resprot.3896)
Supplement: Multimedia Appendix 1 [file resprot_v4i3e86_app1.pdf]

Active-Q – A Web-based Physical Activity Questionnaire for Adults

The following questions concern your physical activity during the past months.

Occupation status
Do you work or study at the moment? Refers to full time, half time and volunteer work.

❍ Yes ❍ Don´t know
❍ No ❍ Don´t want to answer

Physical activity level – daily occupation
What is your normal activity level at your daily occupation (work, studies or equivalent)?

Mostly sitting	A combination of sitting and standing up	Mostly standing and walking	Some physical activity	Heavy manual labor
▯	▯	▯	▯	▯

Duration - daily occupation
Approximately how many hours per week do you normally conduct your daily occupation (work, studies or equivalent)?

❍ 5 hours or less
❍ 6 - 10 hours
❍ 11 – 20 hours
❍ 21 – 30 hours
❍ 31 – 40 hours
❍ 41 – 50 hours
❍ More than 50 hours
❍ Don´t know
❍ Don´t want to answer

Means of transportation
How do you normally get to and from work, studies or other daily occupation?

❑ Walking
❑ Cykling
❑ By motorcycle, moped or scooter
❑ By car or taxi
❑ By bus, train, subway or boat
❑ Other
❍ Does not apply to me
❍ Don´t know
❍ Don´t want to answer

Follow-up questions regarding means of transportation.
Selected activities in the previous screening question are presented to the respondent.

How often do you get to your daily occupation in the following ways and approximately how long does it take?

	Number of days per week	Time, one way per day
Walking	❍ 1<br>❍ 2<br>❍ 3<br>❍ 4<br>❍ 5<br>❍ 6<br>❍ 7<br>❍ Don´t know<br>❍ Don´t want to answer	❍ Less than 15 minutes<br>❍ 15 - 29 minutes<br>❍ 30 - 44 minutes<br>❍ 45 - 59 minutes<br>❍ 1 - 2 hours<br>❍ Longer than 2 hours<br>❍ Don´t know<br>❍ Don´t want to answer
Cycling	❍ 1<br>❍ 2<br>❍ 3<br>❍ 4<br>❍ 5<br>❍ 6<br>❍ 7<br>❍ Don´t know<br>❍ Don´t want to answer	❍ Less than 15 minutes<br>❍ 15 - 29 minutes<br>❍ 30 - 44 minutes<br>❍ 45 - 59 minutes<br>❍ 1 - 2 hours<br>❍ Longer than 2 hours<br>❍ Don´t know<br>❍ Don´t want to answer
By motorcycle, moped or scooter	❍ 1<br>❍ 2<br>❍ 3<br>❍ 4<br>❍ 5<br>❍ 6<br>❍ 7<br>❍ Don´t know<br>❍ Don´t want to answer)	❍ Less than 15 minutes<br>❍ 15 - 29 minutes<br>❍ 30 - 44 minutes<br>❍ 45 - 59 minutes<br>❍ 1 - 2 hours<br>❍ Longer than 2 hours<br>❍ Don´t know<br>❍ Don´t want to answer
By car or taxi	❍ 1<br>❍ 2<br>❍ 3<br>❍ 4<br>❍ 5<br>❍ 6<br>❍ 7<br>❍ Don´t know<br>❍ Don´t want to answer	❍ Less than 15 minutes<br>❍ 15 - 29 minutes<br>❍ 30 - 44 minutes<br>❍ 45 - 59 minutes<br>❍ 1 - 2 hours<br>❍ Longer than 2 hours<br>❍ Don´t know<br>❍ Don´t want to answer
By bus, train, subway or boat	❍ 1<br>❍ 2<br>❍ 3<br>❍ 4	❍ Less than 15 minutes<br>❍ 15 - 29 minutes<br>❍ 30 - 44 minutes<br>❍ 45 - 59 minutes

	Number of days per week	Time, one way per day
	❍ 5<br>❍ 6<br>❍ 7<br>❍ Don´t know<br>❍ Don´t want to answer	❍ 1 - 2 hours<br>❍ Longer than 2 hours<br>❍ Don´t know<br>❍ Don´t want to answer
Other	❍ 1<br>❍ 2<br>❍ 3<br>❍ 4<br>❍ 5<br>❍ 6<br>❍ 7<br>❍ Don´t know<br>❍ Don´t want to answer	❍ Less than 15 minutes<br>❍ 15 - 29 minutes<br>❍ 30 - 44 minutes<br>❍ 45 - 59 minutes<br>❍ 1 - 2 hours<br>❍ Longer than 2 hours<br>❍ Don´t know<br>❍ Don´t want to answer

Leisure time activities

Which of the following activities do you engage in at least once per week in your leisure time?
Mark all activities applicable.

❑ Watching TV/DVDs etc.
❑ Using the computer, reading e-mails, playing computer games, play station, X-box etc.
❑ Sitting reading, writing, sewing etc.
❑ Playing a musical instrument or active computer games (e.g. Wii)
❑ Doing household chores (e.g. cleaning, laundry, child care, gardening etc.)
❑ Shopping or other errands
❑ Dancing
❑ Walking, Nordic walking, walking a dog (not as transport to daily occupation)
❑ Bicycling (not as transport to daily occupation)
❑ Other

❍ None of the above
❍ Don´t know
❍ Don´t want to answer

Follow-up questions regarding leisure time activities.

Selected activities in the previous screening question are presented to the respondent.

How many days per week do you dedicate time to the following leisure time activities and how much time do you spend per day on the activity?

	Number of days per week	Total time per day
Watching TV/DVDs etc.	❍ 1<br>❍ 2<br>❍ 3<br>❍ 4<br>❍ 5<br>❍ 6<br>❍ 7<br>❍ Don´t know<br>❍ Don´t want to answer	❍ Less than 30 minutes<br>❍ 30-59 minutes<br>❍ 60-119 minutes<br>❍ 2-4 hours<br>❍ 5-8 hours<br>❍ Longer than 8 hours<br>❍ Don´t know<br>❍ Don´t want to answer

	Number of days per week	Total time per day
Using the computer, reading e-mails, playing computer games, play station, X-box etc.	❍ 1<br>❍ 2<br>❍ 3<br>❍ 4<br>❍ 5<br>❍ 6<br>❍ 7<br>❍ Don´t know<br>❍ Don´t want to answer	❍ Less than 30 minutes<br>❍ 30-59 minutes<br>❍ 60-119 minutes<br>❍ 2-4 hours<br>❍ 5-8 hours<br>❍ Longer than 8 hours<br>❍ Don´t know<br>❍ Don´t want to answer
Sitting reading, writing, sewing etc.	❍ 1<br>❍ 2<br>❍ 3<br>❍ 4<br>❍ 5<br>❍ 6<br>❍ 7<br>❍ Don´t know<br>❍ Don´t want to answer	❍ Less than 30 minutes<br>❍ 30-59 minutes<br>❍ 60-119 minutes<br>❍ 2-4 hours<br>❍ 5-8 hours<br>❍ Longer than 8 hours<br>❍ Don´t know<br>❍ Don´t want to answer
Playing a musical instrument or active computer games (e.g. Wii)	❍ 1<br>❍ 2<br>❍ 3<br>❍ 4<br>❍ 5<br>❍ 6<br>❍ 7<br>❍ Don´t know<br>❍ Don´t want to answer	❍ Less than 30 minutes<br>❍ 30-59 minutes<br>❍ 60-119 minutes<br>❍ 2-4 hours<br>❍ 5-8 hours<br>❍ Longer than 8 hours<br>❍ Don´t know<br>❍ Don´t want to answer
Doing household chores (e.g. cleaning, laundry, child care, gardening etc.)	❍ 1<br>❍ 2<br>❍ 3<br>❍ 4<br>❍ 5<br>❍ 6<br>❍ 7<br>❍ Don´t know<br>❍ Don´t want to answer	❍ Less than 30 minutes<br>❍ 30-59 minutes<br>❍ 60-119 minutes<br>❍ 2-4 hours<br>❍ 5-8 hours<br>❍ Longer than 8 hours<br>❍ Don´t know<br>❍ Don´t want to answer
Shopping or other errands	❍ 1<br>❍ 2<br>❍ 3<br>❍ 4<br>❍ 5<br>❍ 6<br>❍ 7<br>❍ Don´t know<br>❍ Don´t want to answer	❍ Less than 30 minutes<br>❍ 30-59 minutes<br>❍ 60-119 minutes<br>❍ 2-4 hours<br>❍ 5-8 hours<br>❍ Longer than 8 hours<br>❍ Don´t know<br>❍ Don´t want to answer

	Number of days per week	Total time per day
Dancing	❍ 1<br>❍ 2<br>❍ 3<br>❍ 4<br>❍ 5<br>❍ 6<br>❍ 7<br>❍ Don´t know<br>❍ Don´t want to answer	❍ Less than 30 minutes<br>❍ 30-59 minutes<br>❍ 60-119 minutes<br>❍ 2-4 hours<br>❍ 5-8 hours<br>❍ Longer than 8 hours<br>❍ Don´t know<br>❍ Don´t want to answer
Walking, Nordic walking, walking a dog (not as transport to daily occupation)	❍ 1<br>❍ 2<br>❍ 3<br>❍ 4<br>❍ 5<br>❍ 6<br>❍ 7<br>❍ Don´t know<br>❍ Don´t want to answer	❍ Less than 15 minutes<br>❍ 15-29 minutes<br>❍ 30-44 minutes<br>❍ 45-59 minutes<br>❍ 1-2 hours<br>❍ Longer than 2 hours<br>❍ Don´t know<br>❍ Don´t want to answer
Bicycling (not as transport to daily occupation)	❍ 1<br>❍ 2<br>❍ 3<br>❍ 4<br>❍ 5<br>❍ 6<br>❍ 7<br>❍ Don´t know<br>❍ Don´t want to answer	❍ Less than 15 minutes<br>❍ 15-29 minutes<br>❍ 30-44 minutes<br>❍ 45-59 minutes<br>❍ 1-2 hours<br>❍ Longer than 2 hours<br>❍ Don´t know<br>❍ Don´t want to answer

Overall sporting activity or exercise

Do you exercise or participate in any sporting activities regularly?

❍ Yes ❍ Don´t know
❍ No ❍ Don´t want to answer

Sporting activities

What kind of exercise or sports activities do you participate in? If your type of activity is not included in the list below, please choose the most similar one.

❑ Aerobics or cardio fitness class
❑ Weight lifting
❑ Jogging, running or orienteering
❑ Athletics, (e.g. high jump, long jump or three-step)
❑ Spinning or cycling in demanding terrain
❑ Swimming
❑ Martial arts (e.g. judo or karate)
❑ Boxing or wrestling
❑ Yoga, Pilates or Tai chi
❑ Tennis, badminton or table tennis
❑ Squash
❑ Sailing, surfing, canoeing or rowing

❑ Ball sports in team (e.g. soccer, basketball, volleyball or floorball)
❑ Golf
❑ Dance class or competitive dancing
❑ Horseback riding
❑ Ice skating, ice hockey or bandy
❑ Skiing downhill or cross country
❑ Motor sports (e.g. motorcross)
❑ Rock climbing
❑ Other

❍ None of these
❍ Don´t know
❍ Don´t want to answer

Follow-up questions regarding regular sporting activities.

Selected activities in the previous screening question are presented to the respondent.

How often do you participate in the following sports or the following activities and for how long each time?

	Number of times	Time per session
Aerobics or cardio fitness class	❍ 1 - 3 times a month<br>❍ Once a week<br>❍ 2 - 3 times a week<br>❍ 4 - 5 times a week<br>❍ 6 - 7 times a week<br>❍ Don´t know<br>❍ Don´t want to answer	❍ Less than 30 minutes<br>❍ 30 - 59 minutes<br>❍ 60 - 119 minutes<br>❍ 2 - 4 hours<br>❍ More than 4 hours<br>❍ Don´t know<br>❍ Don´t want to answer
Weight lifting	❍ 1 - 3 times a month<br>❍ Once a week<br>❍ 2 - 3 times a week<br>❍ 4 - 5 times a week<br>❍ 6 - 7 times a week<br>❍ Don´t know<br>❍ Don´t want to answer	❍ Less than 30 minutes<br>❍ 30 - 59 minutes<br>❍ 60 - 119 minutes<br>❍ 2 - 4 hours<br>❍ More than 4 hours<br>❍ Don´t know<br>❍ Don´t want to answer
Jogging, running or orienteering	❍ 1 - 3 times a month<br>❍ Once a week<br>❍ 2 - 3 times a week<br>❍ 4 - 5 times a week<br>❍ 6 - 7 times a week<br>❍ Don´t know<br>❍ Don´t want to answer	❍ Less than 30 minutes<br>❍ 30 - 59 minutes<br>❍ 60 - 119 minutes<br>❍ 2 - 4 hours<br>❍ More than 4 hours<br>❍ Don´t know<br>❍ Don´t want to answer
Athletics, (e.g. high jump, long jump or three-step)	❍ 1 - 3 times a month<br>❍ Once a week<br>❍ 2 - 3 times a week<br>❍ 4 - 5 times a week<br>❍ 6 - 7 times a week<br>❍ Don´t know<br>❍ Don´t want to answer	❍ Less than 30 minutes<br>❍ 30 - 59 minutes<br>❍ 60 - 119 minutes<br>❍ 2 - 4 hours<br>❍ More than 4 hours<br>❍ Don´t know<br>❍ Don´t want to answer
Spinning or cycling in demanding terrain	❍ 1 - 3 times a month<br>❍ Once a week<br>❍ 2 - 3 times a week	❍ Less than 30 minutes<br>❍ 30 - 59 minutes<br>❍ 60 - 119 minutes

	Number of times	Time per session
	❍ 4 - 5 times a week<br>❍ 6 - 7 times a week<br>❍ Don´t know<br>❍ Don´t want to answer	❍ 2 - 4 hours<br>❍ More than 4 hours<br>❍ Don´t know<br>❍ Don´t want to answer
Swimming	❍ 1 - 3 times a month<br>❍ Once a week<br>❍ 2 - 3 times a week<br>❍ 4 - 5 times a week<br>❍ 6 - 7 times a week<br>❍ Don´t know<br>❍ Don´t want to answer	❍ Less than 30 minutes<br>❍ 30 - 59 minutes<br>❍ 60 - 119 minutes<br>❍ 2 - 4 hours<br>❍ More than 4 hours<br>❍ Don´t know<br>❍ Don´t want to answer
Ball sports in team (e.g. soccer, basketball, voleyball or floorball	❍ 1 - 3 times a month<br>❍ Once a week<br>❍ 2 - 3 times a week<br>❍ 4 - 5 times a week<br>❍ 6 - 7 times a week<br>❍ Don´t know<br>❍ Don´t want to answer	❍ Less than 30 minutes<br>❍ 30 - 59 minutes<br>❍ 60 - 119 minutes<br>❍ 2 - 4 hours<br>❍ More than 4 hours<br>❍ Don´t know<br>❍ Don´t want to answer
Golf	❍ 1 - 3 times a month<br>❍ Once a week<br>❍ 2 - 3 times a week<br>❍ 4 - 5 times a week<br>❍ 6 - 7 times a week<br>❍ Don´t know<br>❍ Don´t want to answer	❍ Less than 30 minutes<br>❍ 30 - 59 minutes<br>❍ 60 - 119 minutes<br>❍ 2 - 4 hours<br>❍ More than 4 hours<br>❍ Don´t know<br>❍ Don´t want to answer
Dance class or competitive dancing	❍ 1 - 3 times a month<br>❍ Once a week<br>❍ 2 - 3 times a week<br>❍ 4 - 5 times a week<br>❍ 6 - 7 times a week<br>❍ Don´t know<br>❍ Don´t want to answer	❍ Less than 30 minutes<br>❍ 30 - 59 minutes<br>❍ 60 - 119 minutes<br>❍ 2 - 4 hours<br>❍ More than 4 hours<br>❍ Don´t know<br>❍ Don´t want to answer
Horseback riding	❍ 1 - 3 times a month<br>❍ Once a week<br>❍ 2 - 3 times a week<br>❍ 4 - 5 times a week<br>❍ 6 - 7 times a week<br>❍ Don´t know<br>❍ Don´t want to answer	❍ Less than 30 minutes<br>❍ 30 - 59 minutes<br>❍ 60 - 119 minutes<br>❍ 2 - 4 hours<br>❍ More than 4 hours<br>❍ Don´t know<br>❍ Don´t want to answer
Ice skating, ice hockey or bandy	❍ 1 - 3 times a month<br>❍ Once a week<br>❍ 2 - 3 times a week<br>❍ 4 - 5 times a week<br>❍ 6 - 7 times a week<br>❍ Don´t know	❍ Less than 30 minutes<br>❍ 30 - 59 minutes<br>❍ 60 - 119 minutes<br>❍ 2 - 4 hours<br>❍ More than 4 hours<br>❍ Don´t know

	Number of times	Time per session
	❍ Don´t want to answer	❍ Don´t want to answer
Skiing downhill or cross country	❍ 1 - 3 times a month<br>❍ Once a week<br>❍ 2 - 3 times a week<br>❍ 4 - 5 times a week<br>❍ 6 - 7 times a week<br>❍ Don´t know<br>❍ Don´t want to answer	❍ Less than 30 minutes<br>❍ 30 - 59 minutes<br>❍ 60 - 119 minutes<br>❍ 2 - 4 hours<br>❍ More than 4 hours<br>❍ Don´t know<br>❍ Don´t want to answer
Martial arts (e.g. judo or karate)	❍ 1 - 3 times a month<br>❍ Once a week<br>❍ 2 - 3 times a week<br>❍ 4 - 5 times a week<br>❍ 6 - 7 times a week<br>❍ Don´t know<br>❍ Don´t want to answer	❍ Less than 30 minutes<br>❍ 30 - 59 minutes<br>❍ 60 - 119 minutes<br>❍ 2 - 4 hours<br>❍ More than 4 hours<br>❍ Don´t know<br>❍ Don´t want to answer
Boxing or wrestling	❍ 1 - 3 times a month<br>❍ Once a week<br>❍ 2 - 3 times a week<br>❍ 4 - 5 times a week<br>❍ 6 - 7 times a week<br>❍ Don´t know<br>❍ Don´t want to answer	❍ Less than 30 minutes<br>❍ 30 - 59 minutes<br>❍ 60 - 119 minutes<br>❍ 2 - 4 hours<br>❍ More than 4 hours<br>❍ Don´t know<br>❍ Don´t want to answer
Yoga, Pilates or Tai chi	❍ 1 - 3 times a month<br>❍ Once a week<br>❍ 2 - 3 times a week<br>❍ 4 - 5 times a week<br>❍ 6 - 7 times a week<br>❍ Don´t know<br>❍ Don´t want to answer	❍ Less than 30 minutes<br>❍ 30 - 59 minutes<br>❍ 60 - 119 minutes<br>❍ 2 - 4 hours<br>❍ More than 4 hours<br>❍ Don´t know<br>❍ Don´t want to answer)
Tennis, badminton or table tennis	❍ 1 - 3 times a month<br>❍ Once a week<br>❍ 2 - 3 times a week<br>❍ 4 - 5 times a week<br>❍ 6 - 7 times a week<br>❍ Don´t know<br>❍ Don´t want to answer	❍ Less than 30 minutes<br>❍ 30 - 59 minutes<br>❍ 60 - 119 minutes<br>❍ 2 - 4 hours<br>❍ More than 4 hours<br>❍ Don´t know<br>❍ Don´t want to answer
Squash	❍ 1 - 3 times a month<br>❍ Once a week<br>❍ 2 - 3 times a week<br>❍ 4 - 5 times a week<br>❍ 6 - 7 times a week<br>❍ Don´t know<br>❍ Don´t want to answer	❍ Less than 30 minutes<br>❍ 30 - 59 minutes<br>❍ 60 - 119 minutes<br>❍ 2 - 4 hours<br>❍ More than 4 hours<br>❍ Don´t know<br>❍ Don´t want to answer
Sailing, surfing, canoeing or rowing	❍ 1 - 3 times a month<br>❍ Once a week	❍ Less than 30 minutes<br>❍ 30 - 59 minutes

	Number of times	Time per session
	❍ 2 - 3 times a week<br>❍ 4 - 5 times a week<br>❍ 6 - 7 times a week<br>❍ Don´t know<br>❍ Don´t want to answer	❍ 60 - 119 minutes<br>❍ 2 - 4 hours<br>❍ More than 4 hours<br>❍ Don´t know<br>❍ Don´t want to answer
Motor sports (e.g. motorcross)	❍ 1 - 3 times a month<br>❍ Once a week<br>❍ 2 - 3 times a week<br>❍ 4 - 5 times a week<br>❍ 6 - 7 times a week<br>❍ Don´t know<br>❍ Don´t want to answer	❍ Less than 30 minutes<br>❍ 30 - 59 minutes<br>❍ 60 - 119 minutes<br>❍ 2 - 4 hours<br>❍ More than 4 hours<br>❍ Don´t know<br>❍ Don´t want to answer
Rock climbing	❍ 1 - 3 times a month<br>❍ Once a week<br>❍ 2 - 3 times a week<br>❍ 4 - 5 times a week<br>❍ 6 - 7 times a week<br>❍ Don´t know<br>❍ Don´t want to answer	❍ Less than 30 minutes<br>❍ 30 - 59 minutes<br>❍ 60 - 119 minutes<br>❍ 2 - 4 hours<br>❍ More than 4 hours<br>❍ Don´t know<br>❍ Don´t want to answer

Competing

Do you compete regularly in a sport? Follow-up question if respondent reported regular physical activity.

❍ Yes ❍ Don´t know
❍ No ❍ Don´t want to answer

Sleep

How many hours do you normally sleep per night?

❍ Less than 5 hours ❍ 9 hours
❍ 6 hours ❍ 10 hours or more
❍ 7 hours ❍ Don´t know
❍ 8 hours ❍ Don´t want to answer
